# Spatial correlation of cell stiffness and traction forces in cancer cells measured with combined SICM and TFM†

**DOI:** 10.1039/d1ra01277k

**Published:** 2021-04-14

**Authors:** Johannes Rheinlaender, Hannes Wirbel, Tilman E. Schäffer

**Affiliations:** Institute of Applied Physics, University of Tübingen Auf der Morgenstelle 10 72076 Tübingen Germany johannes.rheinlaender@uni-tuebingen.de tilman.schaeffer@uni-tuebingen.de +49 7071 29 5093 +49 7071 29 76030

## Abstract

The mechanical properties of cancer cells at the single-cell and the subcellular level might be the key for answering long-standing questions in the diagnosis and treatment of cancer. However, the subcellular distribution of two main mechanical properties, cell stiffness and traction forces, has been investigated only rarely and qualitatively yet. Here, we present the first direct combination of scanning ion conductance microscopy (SICM) and traction force microscopy (TFM), which we used to identify a correlation between the local stiffness and the local traction force density in living cells. We found a correlation in normal breast epithelial cells, but no correlation in cancerous breast epithelial cells. This indicates that the interplay between cell stiffness and traction forces is altered in cancer cells as compared to healthy cells, which might give new insight in the research field of cancer cell mechanobiology.

## Abstract 2

Using combined SICM and TFM we identified a subcellular correlation between the local stiffness and traction force density in living cells. We found this correlation in normal breast epithelial cells, but not in cancerous breast epithelial cells.

## Graphical abstract

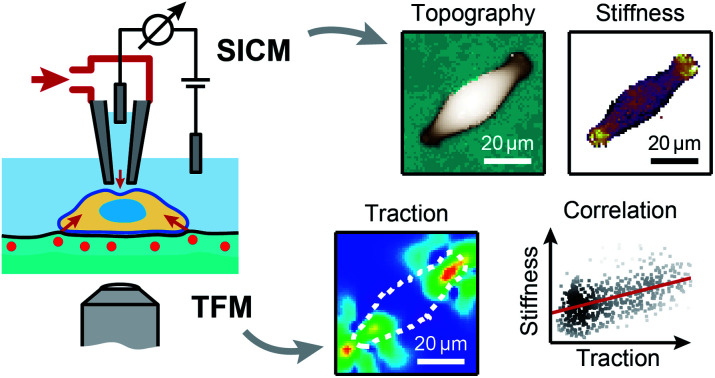

## Introduction

The mechanical properties of cancer cells at the single-cell level are an important aspect in the understanding of cancer.^1^ Many studies have shown that cancer cells differ in their mechanical stiffness from their healthy or “normal” phenotype, as for example measured with an optical stretcher,^2^ atomic force microscopy (AFM),^3,4^ microfluidic devices,^5^ intracellular particle tracking,^6^ magnetic tweezer,^7^ or micropipette aspiration.^8^ However, there is no general consensus^9^ whether cancer cells are softer^2–7,10–15^ or stiffer^8,16–18^ than normal cells.

This might be due to the fact that another mechanical property is often unappreciated: the contractile forces actively generated by the cell.^19^ Recently, we have identified a correlation between “active” cellular traction forces and “passive” cell stiffness on the cell-to-cell level^20^ and there are hints for higher traction forces in cancer cells than in normal cells^21–24^ but also the contrary.^25^ However, the influence of cellular traction forces in the context of cancer mechanobiology has only rarely been investigated yet.^26^

In the present study, we used a unique experimental setup to investigate both the passive mechanical properties and the active contraction forces on the single-cell level and applied it to normal and cancerous breast epithelial cells. By combining scanning ion conductance microscopy (SICM),^27,28^ a specialized scanning probe microscopy (SPM) technique for imaging^29^ and mechanical investigation of live cells,^30^ and traction force microscopy (TFM),^19^ we were able to simultaneously measure mechanical stiffness and traction forces of living cells with subcellular resolution, thereby allowing to directly correlate these quantities. Combined SICM-TFM demonstrated that increased traction forces are accompanied by higher cell stiffness, resulting in a correlation of local stiffness and traction forces. While we found a correlation in normal MCF10A breast epithelial cells, a correlation was usually not observed in cancerous MCF7 breast epithelial cells, which might be an important, yet so far unknown aspect of cancer cell mechanobiology.

## Results

### Combined SICM and TFM of living cells

**Fig. 1:**
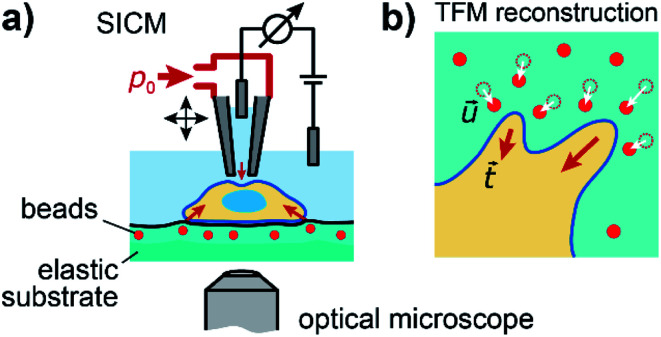
Combined SICM and TFM of living cells. (a) Schematic of the combined SICM (top) and TFM (bottom) setup. A nanopipette with pressure *p*_0_ applied to its upper end is approached to a living cell, which is adhered to an elastic substrate with embedded fluorescent marker beads. (b) Schematic of TFM reconstruction of cellular traction forces 
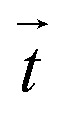
 (red arrows) from substrate displacement 
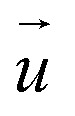
 (white arrows).

To investigate the relation between mechanical stiffness and traction forces in living cells, we combined SICM and TFM within one experimental setup (Fig. 1a, for details see Methods). For TFM, the cells were grown on elastomer substrates with embedded fluorescent marker beads, allowing the reconstruction of cellular traction forces from the substrate displacement (Fig. 1b, for details see Methods).

### Large cell stiffness coincides with large traction forces

**Fig. 2:**
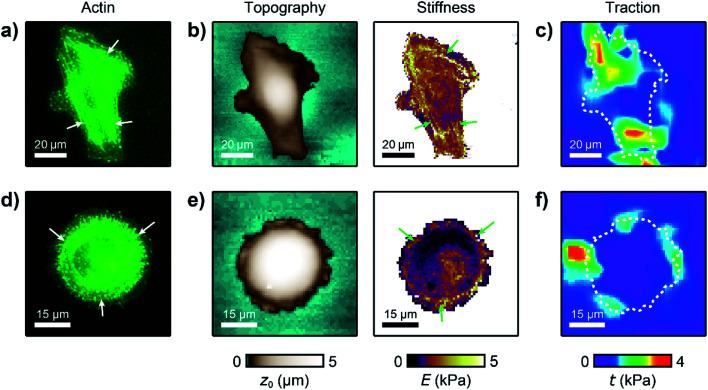
Large cell stiffness coincides with large traction forces in living U2OS cells. (a) Actin fluorescence intensity, (b) SICM topography image (left) and stiffness map (right), and (c) traction force density of a polarized U2OS cell. (d) Actin fluorescence, (e) SICM topography image (left) and stiffness map (right), and (f) traction force density of an unpolarized U2OS cell. The dashed lines in (c) and (f) outline the cell contour.

**Fig. 3:**
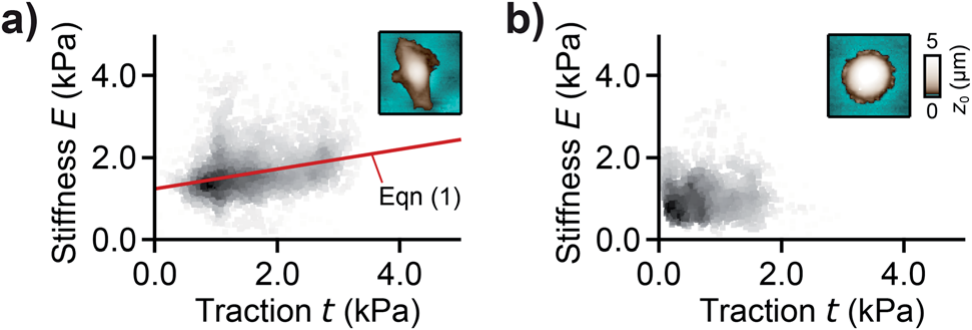
Correlation between local stiffness and traction. (a) Local stiffness as a function of local traction force density for the cell shown in Fig. 2a–c and (b) for the cell shown in Fig. 2d–f. The red line is a fit of eqn (1). The scatter plot grayscale level indicates point density. The insets show the respective SICM topography images of the cells from Fig. 2. Number of data points 2835 (a) and 1277 (b).

For analyzing the relation between cell stiffness and traction forces, we investigated U2OS cells that express GFP-labeled actin. A well-spread, polarized U2OS cell showed dominant parallel stress fibers (Fig. 2a, white arrows) along its long axis. Unfortunately, due to their broad fluorescence spectrum, the fluorescent marker beads were also visible in the actin fluorescence images due to bleed-through. In SICM, the cell showed an elongated morphology in the topography image (Fig. 2b, left) and a soft cell body and a stiff cell periphery in the stiffness map (Fig. 2b, right). Individual stress fibers can be identified (Fig. 2b, green arrows). A less spread, unpolarized U2OS cell showed fewer stress fibers but a dendritic actin cytoskeleton in the cell periphery (Fig. 2d, white arrows). In the SICM topography image, the cell exhibited a round morphology (Fig. 2e, left). The stiffness map (Fig. 2e, right) revealed a soft cell body and a slightly stiffer periphery (Fig. 2e, green arrows). In TFM, the cells showed different traction force distributions. For the polarized cell there are regions of high traction forces (Fig. 2c, green to red colors), which coincided with regions of high cell stiffness (Fig. 2b, yellow to white colors), and *vice versa*. Consequently, the local stiffness was correlated positively with local traction force density (Fig. 3a, *ρ* = 0.29 ± 0.02, *P* < 10^−10^). The local stiffness *E* and the traction force density *t* followed an approximately linear relationship

1 *E*(*t*) = *E*_0_ + *m × t*

with a stiffness baseline *E*_0_ on the order of 1 kPa and a dimensionless factor *m* typically between 0.1 and 0.4 (here *E*_0_ = 1.2 kPa and *m* = 0.24). In the case of the unpolarized cell, the traction forces were generally lower (Fig. 2f) and no correlation was found between local stiffness and traction forces (Fig. 3b, *ρ* = −0.01 ± 0.03, *P* = 0.6).

### Combined SICM and TFM of normal and cancerous human breast epithelial cells

**Fig. 4:**
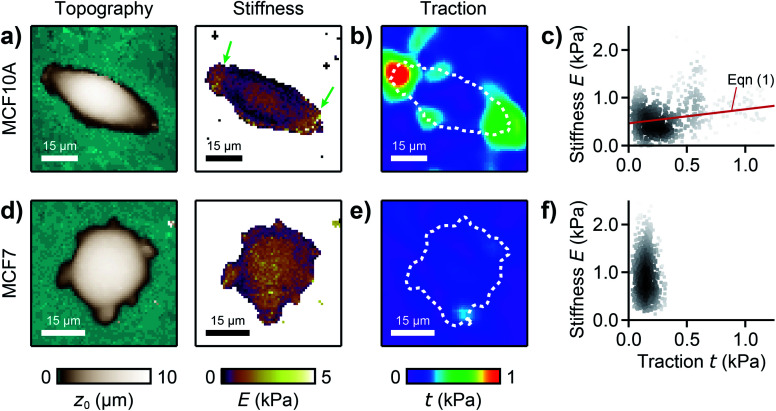
Combined SICM and TFM of normal and cancerous human breast epithelial cells. (a) SICM topography image (left) and stiffness map (right), (b) traction force density, and (c) local stiffness as a function of traction of a normal MCF10A human breast epithelial cell. (d) SICM topography image (left) and stiffness map (right), (e) traction force density, and (f) local stiffness as a function of traction of a cancerous MCF7 human breast epithelial cell. The red line is a fit of eqn (1). The dashed lines in (b) and (e) outline the cell contour. The scatter plot grayscale level in (c) and (f) indicates point density. Number of data points 951 (c) and 1228 (f).

We used our combined SICM and TFM approach to investigate MCF10A and MCF7 human breast epithelial cells (Fig. 4, see Methods for details), which are widely-used model systems for normal epithelial and breast cancer cells, respectively,^31^ and are known to differ in morphology, mechanical stiffness,^32^ and contractile forces.^24^ MCF10A cells exhibited an elongated morphology, a soft cell body and usually two stiff cell extensions (Fig. 4a, green arrows), where high traction forces were located (Fig. 4b). Consequently, local stiffness was correlated positively with local traction force density (Fig. 4c, *ρ* = 0.19 ± 0.03, *P* = 2.8 × 10^−9^) and followed a linear relationship (here *E*_0_ = 0.47 kPa and *a* = 0.29). In contrast, MCF7 cells showed a less elongated and more rounded morphology with many smaller extensions and a more homogeneous stiffness distribution (Fig. 4d). As TFM also showed a more homogenous traction force distribution (Fig. 4e), no correlation between local stiffness and traction force density was found here (Fig. 4f, *ρ* = 0.03 ± 0.03, *P* = 0.2).

**Fig. 5:**
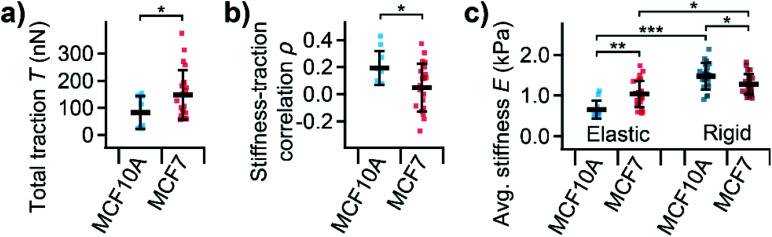
Total traction, subcellular correlation, and average stiffness of normal and cancerous human breast epithelial cells. (a) Total traction *T* and (b) correlation coefficient *ρ* of subcellular correlation between local stiffness and traction for normal MCF10A and cancerous MCF7 human breast epithelial cells. (c) Average stiffness *E* for normal MCF10A and cancerous MCF7 cells on elastic PDMS substrates and rigid cell culture dishes. Plots show average (marker), standard deviation (error bar), individual cells (dots); number of cells *n* = 8 and 23 (a and b) or 11 and 24 on elastic and 66 and 86 on rigid substrates (c) for MCF10A and MCF7, respectively; *P < 0.05, **P < 0.01, ***P < 0.001 from Student's *t*-tests (a and b) and Tukey's range test (c).

On average, MCF10A cells showed a significantly lower total traction (Fig. 5a, *P* = 0.039) than MCF7 cells. Furthermore, normal MCF10A cells exhibited a significantly (*P* = 0.010) higher positive correlation between local stiffness and traction force density with *ρ* between 0.1 and 0.4 compared to cancerous MCF7 cells with *ρ* around 0 (Fig. 5b). On elastic PDMS substrates, MCF10A cells were significantly softer than MCF7 cells (Fig. 5c, *P* = 0.0026). On rigid cell culture dishes, both cells types showed a similar morphology (ESI Fig. S-1†), but were generally stiffer than on elastic substrates (Fig. 5c, *P* = 7 × 10^−11^, *P* = 0.012). On rigid substrates, MCF10A cells were stiffer than MCF7 cells (Fig. 5c, *P* = 0.021), which is the opposite behavior than on soft substrates.

## Discussion

We presented the first direct combination of SICM and TFM (Fig. 1), which we applied to study the relation between cell stiffness and traction forces in U2OS cells and normal MCF10A and cancerous MCF7 breast epithelial cells. The combination is technically relatively straightforward as most SICM setups are equipped with an inverted optical microscope, but instead of the commonly used hydrogel TFM substrates we used PDMS elastomer substrates,^33^ as SICM does not work properly on hydrogel samples. This drawback does not exist when combining TFM with AFM.^20^

We corrected for the effect of finite cell thickness on the measured stiffness using a recently introduced model, which assumes the cell being supported by an infinitely stiff substrate.^34^ Although this assumption is, strictly speaking, not fulfilled here, it is nevertheless valid to make this simplification, because the substrates are stiffer than both the apparent and the corrected cell stiffness, and because the correlation between cell stiffness and traction force density is not seriously affected by the correction (ESI Fig. S-2†). The common assumption that the cell is a homogenous elastic structure is certainly not representing the complex real structure of the cytoskeleton,^35^ but promising theoretical models will probably yield more insight into the connection between the experimentally accessible mechanical properties of the cell and its structural components.^36,37^

We demonstrated that areas of large cell stiffness as measured by SICM correlate to the actin density and coincide with regions of high traction forces (Fig. 2), resulting in a spatial correlation of stiffness and traction forces (Fig. 3). For the average cell stiffness, a correlation with traction forces has already been reported,^20,38–40^ which is usually explained by nonlinear stress-stiffening of the actin cytoskeleton.^41–43^ For the local stiffness, however, a spatial correlation with traction forces has not yet been shown, to our knowledge. The interpretation of this correlation is still unclear, but might be caused by the dissipation of contractile forces,^44^ postulated as the “missing piece in cell mechanics”.^45^ In this picture, the cell stiffness would be large at regions of high traction forces due to stress-stiffening, but decreases with larger distance from the regions of high traction forces due to their spatial dissipation, which would explain the correlation observed by us.

We then applied combined SICM and TFM to normal MCF10A and cancerous MCF7 cells (Fig. 4), which are well-studied model systems for normal breast epithelial cells and breast cancer cells, respectively.^31^ The total traction forces in our study were similar to those measured using hydrogel TFM for the same cell line,^21^ consistent with the literature.^24^ We found that MCF7 cancer cells generate higher traction forces compared to their normal MCF10A “counterparts” (Fig. 5a). But, interestingly, we found a correlation between local stiffness and traction forces in normal MCF10A but not in cancerous MCF7 cells (Fig. 5b). This indicates that MCF10A exhibit the “normal” stress-stiffening behavior, while MCF7 cells do not, which might be a particular property of cancer cells, consistent with a more disordered cytoskeleton in cancer cells.^12,22^

On rigid substrates, MCF10A cells were stiffer than MCF7 cells (Fig. 5c), in line with the literature.^6,28^ On elastic substrates, however, normal MCFA10 cells were softer than cancerous MCF7 cells (Fig. 5c). The same behavior was reported for normal and cancerous thyroid and renal cells.^46,47^ It was hypothesized that the stress-stiffening behavior of the cytoskeleton causes the increase of cell stiffness with substrate stiffness.^48^ Our results therefore give more evidence for a fundamental difference in mechanotransduction for normal and cancerous cells on the single-cell level.^26^ This might also be linked to the multicellular level, where cell–cell interactions were found to affect stress-stiffening^32^ and cell migration of individual cells^49^ and confluent monolayers^50^ differently for normal and cancer cells.

In summary, our work shines light on the complex interplay between cell stiffness and contractility in the context of cancer cell mechanobiology, where additional insight may aid answering long-standing questions in disease progression^51^ and might allow to develop new approaches in cancer diagnostics and therapies.^52^

## Methods

### Experimental setup

SICM topography imaging and stiffness mapping was performed with a custom-build setup (for details see ref. 53). The nanopipettes used were pulled from borosilicate glass capillaries (1B100F-4, World Precision Instruments Inc., Sarasota, FL, USA) using a commercial CO_2_-laser-based micropipette puller (P-2000, Sutter Instruments, Novato, CA, USA) and had typical inner opening radii of 200−300 nm. SICM stiffness maps were recorded as described previously.^54^ Briefly, a constant pressure of *p*_0_ = 5 or 10 kPa was applied to the upper end of the capillary and *IZ*-curves were recorded on a raster-pattern across the sample with a typical resolution of 1 μm per pixel. The sample stiffness in terms of the apparent Young's modulus *E*_app_ was then obtained from the slope of the *IZ*-curve between 98 and 99% relative ion current (ESI Material, eqn S1†).^54^ In the stiffness calculation, the substrates were assumed as infinitely stiff for simplicity, as the cells were much (≈10×) softer than the substrates, which might lead into only a small underestimation of cell stiffness. The effect of the finite cell thickness on the measured cell stiffness *E* was corrected as described previously (ESI Material and Fig. S-2†),^34^ assuming cells being incompressible and rigidly bound to the substrate. For the combination with TFM, the SICM setup was mounted on an inverted optical microscope (Ti-U, Nikon, Tokio, Japan) with phase-contrast and epi-fluorescence illumination. Optical images were recorded with a 40×/0.6 NA objective (MRH48430, Nikon) and a high sensitivity monochrome camera (DS-Qi2, Nikon).

### Substrate preparation

TFM substrates from polydimethylsiloxane (PDMS) elastomers were fabricated using a protocol modified after ref. 55. Briefly, compliant PDMS (Gel-8100, NuSil Technology, Carpinteria, CA) was prepared by adding parts A and B at weight ratio 1 : 1 and mixing for 15 min at 1000 rpm using a magnetic stirrer. Then, 40 μL of the mixture was dropped on glass-bottom cell culture dish (81218, Ibidi GmbH Gräfelfing, Germany) and spin-coated (3 s ramp, 10 rps, dwell 6 s), followed by curing for 12 h at 80 °C, yielding a PDMS substrate with reproducible substrate thickness of 100 μm, as verified by confocal microscopy (not shown). Afterwards, a second thin PDMS layer containing fluorescent marker beads was added to the substrate surface. For that, powder of monodisperse fluorescent melamine resin beads (MF-FluoOrange, diameter 934 ± 50 nm, microParticles GmbH, Berlin, Germany) was mixed at weight ratio 1 : 100 with uncured PDMS, stirred for 15 min at 1000 rpm, and sonicated for 15 min at 20 kHz. Then 8 μL of the beads–PDMS mixture was dropped on the cured substrate, spin-coated (10 s ramp, 80 rps, dwell 20 s), and cured for 12 h at 80 °C, yielding a few micrometer thin layer with fluorescent beads with typically 0.1–0.2 beads per μm^2^ (not shown). Finally, the TFM substrates were coated with 0.01% poly-l-lysine (P4707, Sigma-Aldrich, St. Louis, MO) for 24 h at 37 °C to facilitate cell adhesion. As cured PDMS substrates exhibit mainly elastic material properties,^33^ the Young's modulus of the completed substrates was measured as 15 x 1.2 kPa (geometric mean x geometric standard error) using SICM stiffness mapping (17 maps on 12 gels).

### Cell culture

U2OS cells (BioCat GmbH, Heidelberg, Germany), stably expressing GFP-labeled actin, were cultured in DMEM (Biochrom GmbH, Berlin, Germany) supplemented with 10% fetal bovine serum (Biochrom), 2 mM l-Alanyl-l-glutamine (Biochrom), 1% non-essential amino acids (Biochrom), and 100 U ml^−1^ penicillin–streptomycin (Biochrom). MCF10A human mammary epithelial cells^56^ (CRL-10317, ATCC) were cultured in DMEM/Ham's F12 medium with stable glutamine (Biochrom) supplemented with 5% horse-serum, 0.5 μg mL^−1^ hydrocortisone, 5 μg mL^−1^ insulin and 20 ng mL^−1^ epidermal growth factor (Sigma-Aldrich). MCF7 mammary human breast cancer cells^57^ (HTB-22, ATCC) were cultured in MEM (Eagle) medium with stable glutamine (Biochrom), supplemented with 10% fetal calf serum, 1% penicillin/streptomycin (Biochrom), and 1% non-essential amino acid (Biochrom). The cell lines were maintained at 37 °C in a 5% CO_2_ humidified atmosphere. The cells were seeded on TFM substrates (Fig. 2 and 4) or on cell culture dishes (627160, Greiner Bio-One, Kremsmünster, Austria) (ESI Fig. S-1†) at a sparse density 24 h before the measurements. Prior to the measurements, the cell culture medium was replaced with CO_2_-independent Leibovitz L-15 medium (F1315, Biochrom), containing the same supplements as the respective culture medium. All measurements were performed at 37 °C.

### TFM analysis

For each cell, a “deformed” image was recorded immediately after the SICM measurement and a “reference” image 15 min after detaching the cells with 10× trypsin/EDTA in PBS (L2153, Biochrom). TFM analysis was performed using a custom-written software as previously described.^20^ Briefly, the displacement field 
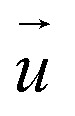
 was calculated from the deformed and reference images by cross-correlation-based particle image velocimetry using an interrogation window of typically 40 × 40 px (containing usually 5−10 beads). From the displacement field the traction force density 
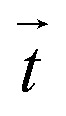
 was calculated using Fourier transform traction cytometry^58^ (FTTC) followed by Gaussian low-pass filtering with 0.01 per px cutoff frequency.

### Statistical analysis

Total traction force *T* was calculated as 
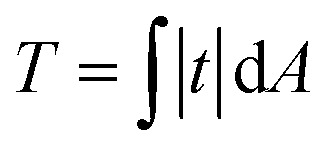
 over the cell area (obtained from the SICM image that was visually aligned with the TFM image) according to.^58^ For robustness against outliers, average cell stiffness was calculated as median, fitting eqn (1) was performed using the Theil-Sen estimator,^59^ and correlation was quantified using Spearman's rank correlation coefficient *ρ* and tested for statistical significance using Spearman's rank correlation test. Differences in average stiffness, total traction, and correlation coefficient were tested for statistical significance using un-paired, two-sided Student's *t*-test or Tukey's range test and indicated using * for *P* ≤ 0.05, ** for *P* ≤ 0.01, and *** for *P* ≤ 0.001. For MCF10A and MCF7 cells, *n* = 8–11 and *n* = 23–24 cells were measured on elastic substrates and *n* = 66 and *n* = 86 cells on rigid substrates, respectively, in *N* = 3 independent experiments each. SICM and TFM data was processed and analyzed using custom-written software in Igor Pro (WaveMetrics Inc., Lake Oswego, OR).

## Conflicts of interest

The authors declare no competing financial interests.

## Supplementary Material

RA-011-D1RA01277K-s001

## References

[cit1] Suresh S. (2007). Acta Biomater..

[cit2] Guck J., Schinkinger S., Lincoln B., Wottawah F., Ebert S., Romeyke M., Lenz D., Erickson H. M., Ananthakrishnan R., Mitchell D., Käs J., Ulvick S., Bilby C. (2005). Biophys. J..

[cit3] Lekka M., Laidler P., Gil D., Lekki J., Stachura Z., Hrynkiewicz A. Z. (1999). Eur. Biophys. J..

[cit4] Cross S. E., Jin Y.-S., Rao J., Gimzewski J. K. (2007). Nat. Nanotechnol..

[cit5] Hou H. W., Li Q. S., Lee G. Y. H., Kumar A. P., Ong C. N., Lim C. T. (2009). Biomed. Microdevices.

[cit6] Li Y., Schnekenburger J., Duits M. (2009). J. Biomed. Opt..

[cit7] Swaminathan V., Mythreye K., O'Brien E. T., Berchuck A., Blobe G. C., Superfine R. (2011). Cancer Res..

[cit8] Pachenari M., Seyedpour S. M., Janmaleki M., Shayan S. B., Taranejoo S., Hosseinkhani H. (2014). J. Biomech..

[cit9] Alibert C., Goud B., Manneville J.-B. (2017). Biol. Cell.

[cit10] Li Q. S., Lee G. Y. H., Ong C. N., Lim C. T. (2008). Biochem. Biophys. Res. Commun..

[cit11] Faria E. C., Ma N., Gazi E., Gardner P., Brown M., Clarke N. W., Snook R. D. (2008). Analyst.

[cit12] Prabhune M., Belge G., Dotzauer A., Bullerdiek J., Radmacher M. (2012). Micron.

[cit13] Ketene A. N., Schmelz E. M., Roberts P. C., Agah M. (2012). Nanomedicine.

[cit14] Coughlin M. F., Bielenberg D. R., Lenormand G., Marinkovic M., Waghorne C. G., Zetter B. R., Fredberg J. J. (2013). Clin. Exp. Metastasis.

[cit15] Yubero M. L., Kosaka P. M., San Paulo Á., Malumbres M., Calleja M., Tamayo J. (2020). Commun. Biol..

[cit16] Zhang G., Long M., Wu Z. Z., Yu W. Q. (2002). World J. Gastroenterol..

[cit17] Rosenbluth M. J., Lam W. A., Fletcher D. A. (2006). Biophys. J..

[cit18] Baker E. L., Lu J., Yu D., Bonnecaze R. T., Zaman M. H. (2010). Biophys. J..

[cit19] Polacheck W. J., Chen C. S. (2016). Nat. Methods.

[cit20] Schierbaum N., Rheinlaender J., Schäffer T. E. (2019). Soft Matter.

[cit21] Kraning-Rush C. M., Califano J. P., Reinhart-King C. A. (2012). PLoS One.

[cit22] Peschetola V., Laurent V. M., Duperray A., Michel R., Ambrosi D., Preziosi L., Verdier C. (2013). Cytoskeleton.

[cit23] Volakis L. I., Li R., Ackerman W. E. I. V., Mihai C., Bechel M., Summerfield T. L., Ahn C. S., Powell H. M., Zielinski R., Rosol T. J., Ghadiali S. N., Kniss D. A. (2014). PLoS One.

[cit24] Li Z., Persson H., Adolfsson K., Abariute L., Borgström M. T., Hessman D., Åström K., Oredsson S., Prinz C. N. (2017). Nanoscale.

[cit25] Koch T. M., Münster S., Bonakdar N., Butler J. P., Fabry B. (2012). PLoS One.

[cit26] Chin L., Xia Y., Discher D. E., Janmey P. A. (2016). Curr. Opin. Chem. Eng..

[cit27] Hansma P. K., Drake B., Marti O., Gould S. A., Prater C. B. (1989). Science.

[cit28] Korchev Y. E., Bashford C. L., Milovanovic M., Vodyanoy I., Lab M. J. (1997). Biophys. J..

[cit29] Happel P., Thatenhorst D., Dietzel I. D. (2012). Sensors.

[cit30] Schäffer T. E. (2013). Anal. Chem..

[cit31] Lacroix M., Leclercq G. (2004). Breast Cancer Res. Treat..

[cit32] Schierbaum N., Rheinlaender J., Schäffer T. E. (2017). Acta Biomater..

[cit33] Style R. W., Boltyanskiy R., German G. K., Hyland C., MacMinn C. W., Mertz A. F., Wilen L. A., Xu Y., Dufresne E. R. (2014). Soft Matter.

[cit34] Rheinlaender J., Schäffer T. E. (2020). Appl. Phys. Lett..

[cit35] Chen J. (2014). Interface Focus.

[cit36] Barreto S., Clausen C. H., Perrault C. M., Fletcher D. A., Lacroix D. (2013). Biomaterials.

[cit37] Yang W., Lacroix D., Tan L. P., Chen J. (2021). J. Mater. Res..

[cit38] Trepat X., Grabulosa M., Puig F., Maksym G. N., Navajas D., Farre R. (2004). Am. J. Physiol. Lung Cell. Mol. Physiol..

[cit39] Kollmannsberger P., Mierke C. T., Fabry B. (2011). Soft Matter.

[cit40] Canović E. P., Seidl D. T., Polio S. R., Oberai A. A., Barbone P. E., Stamenović D., Smith M. L. (2014). Biomech. Model. Mechanobiol..

[cit41] Fernández P., Pullarkat P. A., Ott A. (2006). Biophys. J..

[cit42] Koenderink G. H., Dogic Z., Nakamura F., Bendix P. M., MacKintosh F. C., Hartwig J. H., Stossel T. P., Weitz D. A. (2009). Proc. Natl. Acad. Sci. U. S. A..

[cit43] Kollmannsberger P., Fabry B. (2011). Annu. Rev. Mater. Res..

[cit44] Vignaud T., Copos C., Leterrier C., Toro-Nahuelpan M., Tseng Q., Mahamid J., Blanchoin L., Mogilner A., Théry M., Kurzawa L. (2020). Nat. Mater..

[cit45] Kurzawa L., Vianay B., Senger F., Vignaud T., Blanchoin L., Théry M. (2017). Mol. Biol. Cell.

[cit46] Rianna C., Radmacher M. (2017). Eur. Biophys. J..

[cit47] Rianna C., Radmacher M. (2017). Nanoscale.

[cit48] Solon J., Levental I., Sengupta K., Georges P. C., Janmey P. A. (2007). Biophys. J..

[cit49] Brückner D. B., Arlt N., Fink A., Ronceray P., Rädler J. O., Broedersz C. P. (2021). Proc. Natl. Acad. Sci. U. S. A..

[cit50] Tambe D. T., Corey Hardin C., Angelini T. E., Rajendran K., Park C. Y., Serra-Picamal X., Zhou E. H., Zaman M. H., Butler J. P., Weitz D. A., Fredberg J. J., Trepat X. (2011). Nat. Mater..

[cit51] Gensbittel V., Kräter M., Harlepp S., Busnelli I., Guck J., Goetz J. G. (2021). Dev. Cell.

[cit52] Wirtz D., Konstantopoulos K., Searson P. C. (2011). Nat. Rev. Cancer.

[cit53] Rheinlaender J., Geisse N. A., Proksch R., Schäffer T. E. (2011). Langmuir.

[cit54] Rheinlaender J., Schäffer T. E. (2013). Soft Matter.

[cit55] Yoshie H., Koushki N., Kaviani R., Tabatabaei M., Rajendran K., Dang Q., Husain A., Yao S., Li C., Sullivan J. K., Saint-Geniez M., Krishnan R., Ehrlicher A. J. (2018). Biophys. J..

[cit56] Soule H. D., Maloney T. M., Wolman S. R., Peterson W. D., Brenz R., McGrath C. M., Russo J., Pauley R. J., Jones R. F., Brooks S. C. (1990). Cancer Res..

[cit57] Soule H. D., Vazquez J., Long A., Albert S., Brennan M. (1973). J. Natl. Cancer Inst..

[cit58] Butler J. P., Tolić-Nørrelykke I. M., Fabry B., Fredberg J. J. (2002). Am. J. Physiol. Cell Physiol..

[cit59] Wilcox R. , in Introduction to Robust Estimation and Hypothesis Testing, Academic Press, 2012, pp. 423–427

