# Supplementary material for: Spatial correlation of cell stiffness and traction forces in cancer cells measured with combined SICM and TFM
Source: RSC Adv. 2021 Apr 14;11(23):13951–6. doi: 10.1039/d1ra01277k (PMC8697701; doi:10.1039/d1ra01277k)
Supplement: RA-011-D1RA01277K-s001 [file RA-011-D1RA01277K-s001.pdf]

Electronic Supplementary Material (ESI) for RSC Advances.
This journal is © The Royal Society of Chemistry 2021

Supplemental Material

Spatial Correlation of Cell Stiffness and Traction Forces in Cancer Cells Measured with Combined SICM and TFM

J. Rheinlaender, Hannes Wirbel, and T. E. Schäffer

Institute of Applied Physics, University of Tübingen, Auf der Morgenstelle 10, 72076 Tübingen, Germany.
E-mail: johannes.rheinlaender@uni-tuebingen.de & tilman.schaeffer@uni-tuebingen.de

Cell Morphology and Stiffness on Rigid Substrates

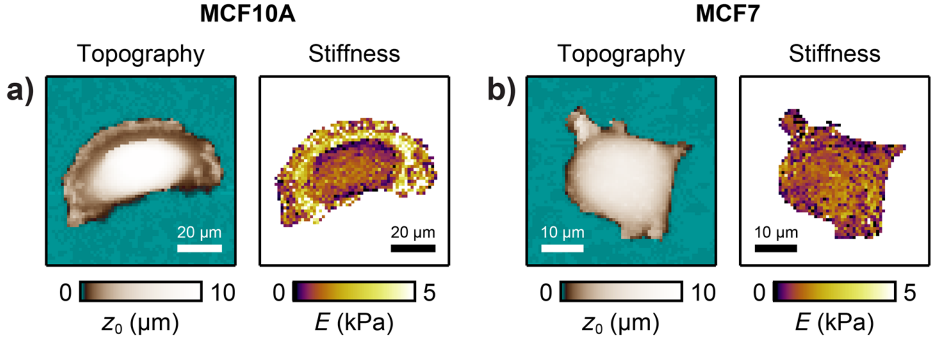

Supplementary Figure S-1 | Cell morphology and stiffness on rigid substrates. (a) SICM topography image (left) and stiffness map (right) of a normal MCF10A and **(b)** of a cancerous MCF7 human breast epithelial cell on rigid cell culture dishes. On rigid substrates, the cells are more spread, but show a similar morphology compared to cells on elastic TFM substrates (see Figure 4).

Theoretical Modell and Effect of Finite Cell Thickness

The apparent cell stiffness E_{app} was measured as described previously.[1] Briefly, a constant pressure p_0 was applied to the upper end of the capillary and *IZ*-curves were recorded. The sample stiffness in terms of the apparent Young's modulus E_{app} was then obtained from the slope s of the *IZ*-curve between 98 and 99% relative ion current as

$$E_{\mathrm{app}} = p_0 A \left(\frac{s_\infty}{s} - 1\right)^{-1}, \tag{S1}$$

where s_∞ is the slope measured on the substrate and $A = 0.26$ is a pipette-dependent geometrical parameter.

As demonstrated for the cell shown in Figure 2b, flat extensions of the cells sometimes appear comparably stiff (also see, e.g., Supplementary Figure S-2a and b). The presence of large traction forces in the extensions might induce an artificial correlation between apparent stiffness and traction force density (Supplementary Figure S-2c, $\rho = 0.12 \pm 0.02$). To avoid this possible artifact, the effect of the finite cell thickness was corrected by[2]

$$E = E_{\mathrm{app}} \cdot \left\{\exp\left(a\frac{r_{\mathrm{i}}}{h}\right) + b\frac{r_{\mathrm{i}}}{h}\left[1 - \exp\left(-c\frac{r_{\mathrm{i}}}{h}\right)\right]\right\}^{-1} \tag{S2}$$

with cell thickness h (here equivalent to the cell height z_0) and pipette inner opening radius r_{i} and using $a = 1.462$, $b = 3.30$, and $c = 0.66$, assuming a Poisson's ratio $\nu = 0.499$ and the cell sticking to an infinitely stiff underlying substrate. The corrected cell stiffness (Supplementary Figure S-2d) is generally lower than the apparent cell stiffness (Supplementary Figure S-2b), for example by a factor of 1.7 for a thickness of $1\ \mu\mathrm{m}$, but still shows a similar subcellular distribution with a soft cell body and stiffer extensions. The correlation between cell stiffness and traction force density is even stronger for the corrected cell stiffness (Supplementary Figure S-1e, $\rho = 0.29 \pm 0.02$). Both the apparent and the corrected cell stiffness (median stiffness here $4.9\ \mathrm{kPa}$ and $1.6\ \mathrm{kPa}$, respectively) are significantly softer than the substrate ($E \cong 15\ \mathrm{kPa}$), indicating that the assumption of an infinitely stiff underlying substrate was valid.

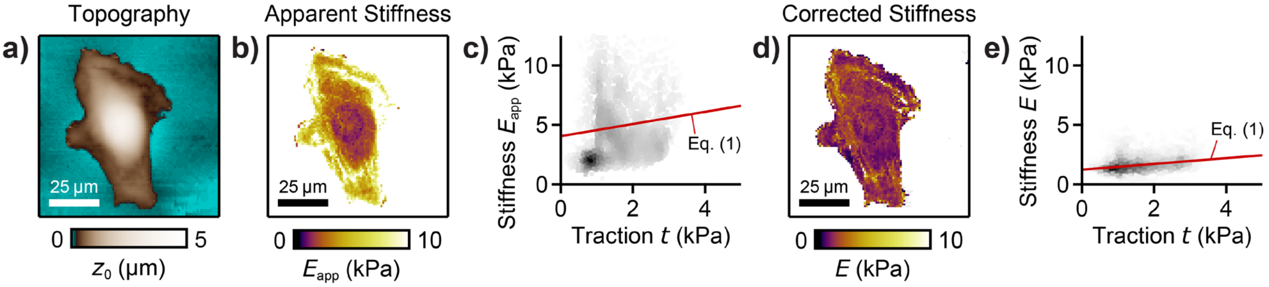

Supplementary Figure S-2 | Effect of cell thickness on measured cell stiffness. (a) SICM topography image, **(b)** map of apparent stiffness, and **(c)** apparent local cell stiffness as a function of local traction force density for the cell shown in Figure 2b and 3a. **(d)** Map of corrected stiffness calculated using Equation (S2) (with $r_{\mathrm{i}} = 300\ \mathrm{nm}$) and **(e)** corrected local cell stiffness as a function of local traction force density. The red lines are fits of Equation (1). The scatter plot grayscale level indicates point density.

References

1. J. Rheinlaender and T. E. Schäffer, *Soft Matter*, 2013, **9**, 3230-3236.
2. J. Rheinlaender and T. E. Schäffer, *Appl. Phys. Lett.*, 2020, **117**, 113701.
